# Sustained Focal Vascular Inflammation Accelerates Atherosclerosis in Remote Arteries

**DOI:** 10.1161/ATVBAHA.120.314387

**Published:** 2020-07-16

**Authors:** Begoña Lavin Plaza, Alkystis Phinikaridou, Marcelo E. Andia, Myles Potter, Silvia Lorrio, Imran Rashid, Rene M. Botnar

**Affiliations:** 1 From the School of Biomedical Engineering and Imaging Sciences, King’s College London, United Kingdom (B.L.P., A.P., M.P., S.L., I.R., R.M.B.); 2 Radiology Department & Millennium Nucleus for Cardiovascular Magnetic Resonance (M.E.A.), Pontificia Universidad Católica de Chile; 3 Escuela de Ingeniería (R.M.B.), Pontificia Universidad Católica de Chile; 4 Case Cardiovascular Research Institute, Case Western Reserve University, Cleveland, OH (I.R.).

## Abstract

Evidence from preclinical and clinical studies has demonstrated that myocardial infarction promotes atherosclerosis progression. The impact of focal vascular inflammation on the progression and phenotype of remote atherosclerosis remains unknown.

**Approach and Results:**

We used a novel *ApoE*^−/−^ knockout mouse model of sustained arterial inflammation, initiated by mechanical injury in the abdominal aorta. Using serial in vivo molecular MRI and ex vivo histology and flow cytometry, we demonstrate that focal arterial inflammation triggered by aortic injury, accelerates atherosclerosis in the remote brachiocephalic artery. The brachiocephalic artery atheroma had distinct histological features including increased plaque size, plaque permeability, necrotic core to collagen ratio, infiltration of more inflammatory monocyte subsets, and reduced collagen content. We also found that arterial inflammation following focal vascular injury evoked a prolonged systemic inflammatory response manifested as a persistent increase in serum IL-6 (interleukin 6). Finally, we demonstrate that 2 therapeutic interventions—pravastatin and minocycline—had distinct anti-inflammatory effects at the plaque and systemic level.

**Conclusions:**

We show for the first time that focal arterial inflammation in response to vascular injury enhances systemic vascular inflammation, accelerates remote atheroma progression and induces plaques more inflamed, lipid-rich, and collagen-poor in the absence of ischemic myocardial injury. This inflammatory cascade is modulated by pravastatin and minocycline treatments, which have anti-inflammatory effects at both plaque and systemic levels that mitigate atheroma progression.

## Abstract 2

Supplemental Digital Content is available in the text.

**Highlights**

- Sustained aortic inflammation, triggered by focal aortic injury, resulted in the formation of larger and more permeable remote atheroma with higher inflammatory cell count, larger necrotic core, and reduced collagen content.
- Sustained aortic inflammation, triggered by focal aortic injury, has systemic ripple effects that promote more advanced remote atheroma via an acute increase of blood inflammatory monocytes, 24 hours following injury, and persistent elevation of serum interleukin IL (interleukin)-6 for up to 3 months following injury.
- Pravastatin and minocycline treatments decrease plaque burden, endothelial permeability, and monocyte count and increase collagen in the remote atheroma. Each treatment had distinct effects on circulating IL-6 and monocytes.

Clinical and experimental data have established both systemic and arterial inflammation as drivers for the progression, destabilization, and rupture of atherosclerotic plaques leading to subsequent cardiovascular events.^1–5^ Recent results from the PESA study (Progression of Early Subclinical Atherosclerosis) demonstrated the presence of arterial inflammation even in plaque-free segments, suggesting that active arterial inflammation may precede the development of atherosclerosis.^6^ Importantly, arterial inflammation is not limited to a single vascular territory but is widespread,^7,8^ leading to the formation of multiple complex coronary plaques^9^ and fissures^10^ in patients with acute coronary syndromes. Considerable evidence also supports the role of systemic inflammation, immunity, or infection in potentiating atherosclerosis and augmenting cardiovascular risk.^1^ Advances in the understanding of the pathways of systemic inflammation and immunity have provided mechanistic insights that connect traditional cardiovascular risk factors to vascular pathophysiology and the formation of atherosclerotic plaques, which is often observed in multiple vascular segments within the same patient.^1,11^ Consequently, biomarkers of inflammation (eg, high-sensitivity C-reactive protein [hsCRP]) have improved the prediction of incident and recurrent cardiovascular events^12–14^ and can help guide administration of anti-lipid^12^ and anti-inflammatory therapies.^12,15,16^ The seminal CANTOS study (Canakinumab Antiinflammatory Thrombosis Outcome Study) recently established that direct anti-inflammatory treatment alleviates the residual inflammatory burden of recurrent cardiovascular events and ultimately improves outcomes in patients with a prior myocardial infarction.^15,16^

Mechanistic studies in mice revealed that myocardial infarction accelerates atherosclerosis via a neuroimmune axis^3^ and endothelial cell activation.^4^ Moreover, the severity of the focal arterial inflammatory response following angioplasty in humans and the extent of mechanical arterial injury in animal models was shown to correlate with the severity of restenosis and neointima hyperplasia.^17–23^ However, whether local vascular inflammation (initiated by focal arterial injury) is sufficient to accelerate remote atherosclerosis and induce changes in plaque phenotype in the absence of myocardial necrosis or ischemic injury, is yet to be elucidated.

In this study, we hypothesize that sustained vascular inflammation in one arterial segment following focal mechanical injury can exacerbate the progression and composition of atheroma remote to the site of injury. Such vascular-to-vascular effects might involve systemic responses that are amenable to therapeutic intervention. We used a novel mouse model of sustained vascular inflammation in the abdominal aorta and performed serial molecular and functional in vivo magnetic resonance imaging (MRI) in addition to ex vivo experiments to (1) investigate the effects of sustained focal vascular inflammation of the aorta on the size, composition, endothelial function, and permeability of remote atheroma; (2) elucidate the potential systemic inflammatory mechanisms involved in this response; and (3) investigate whether pravastatin and minocycline treatment can mitigate the effects of sustained vascular inflammation on the size and composition of the remote atheroma.

## Methods

Data will be available on request from the authors. This study was performed only with males, as it is well known that plaques develop more reproducibly and with less biological variability.^24,25^ Male apolipoprotein knockout mice (*ApoE*^−/−^) at 8 to 10 weeks (n=78) of age were divided into 5 groups: (1) baseline/control group: *ApoE*^−/−^ mice (n=10) were fed a normal laboratory diet for 12 weeks; (2) high-fat diet (HFD) group: *ApoE*^−/−^ mice (n=24) were fed a HFD for 12 weeks containing 21% fat from lard and 0.15% (wt/wt) cholesterol (Special Diet Services, United Kingdom); (3) HFD+injury group: *ApoE*^−/−^ mice (n=24) underwent aortic injury of the abdominal aorta as previously described^17,26^ (Figure I in the Data Supplement) and switched to HFD for 12 weeks the following day; (4) HFD+injury+Prav group: *ApoE*^−/−^ mice (n=10) underwent aortic injury, followed by HFD for 12 weeks and pravastatin treatment administered in drinking water (40 mg/kg per day; Kemprotec Limited, United Kingdom) the following day; (5) HFD+injury+Mino group: *ApoE*^−/−^ mice (n=10) underwent aortic injury, followed by HFD for 12 weeks and minocycline treatment (an antibiotic with proven anti-inflammatory properties)^27–29^ administered in drinking water (3 mg/kg per day, Mylan, United Kingdom) on the following day. The HFD and HFD+injury groups were longitudinally scanned at 4, 8, and 12 weeks after commencement of the experiment to evaluate disease progression. The treatment groups were only assessed at the 12-week time point to evaluate treatment efficacy. The in vivo MRI experiment consisted of 2 MRI sessions separated by 24 hours. During the first session, the abdominal aorta and the brachiocephalic artery (BCA) were imaged 30 minutes after intravenous administration of gadolinium (Gd)-albumin (Ablavar, Lantheus Medical Imaging, North Billerica). During the second imaging session, the same vascular segments were imaged, 2 hours after intravenous administration of Gd-elastin (ESMA, Lantheus Medical Imaging, North Billerica). Following the last imaging session at 12 weeks, tissue, blood, and serum were collected from the animals that underwent MRI plus additional mice per group for ex vivo analysis using histology, flow cytometry, and Luminex assay. In addition, blood was collected from animals at the acute phase following vascular injury (1, 2, and 7 days) to assess the systemic inflammatory response by measuring different cytokines and monocytes. All procedures used in this study were performed in accordance with the guidelines of the UK Home Office. Multiple-group comparisons of continuous variables were performed with a Kruskal-Wallis nonparametric ANOVA test and a Dunn post hoc test (% change of vascular permeability in the aorta and BCA and % change of elastin remodeling and plaque burden in the aorta and BCA, respectively). Correlation analysis was performed with a Spearman test. GraphPad Prism 5.00 (San Diego) was used for the statistical analysis. The data are presented as the media±SEM and *P*<0.05 were considered statistically significant. Detailed methods and power of calculation analysis are described in the material and Figure II in the Data Supplement.

## Results

### Sustained Arterial Inflammation Accelerates Atherosclerosis in a Remote Artery

**Figure 1.**
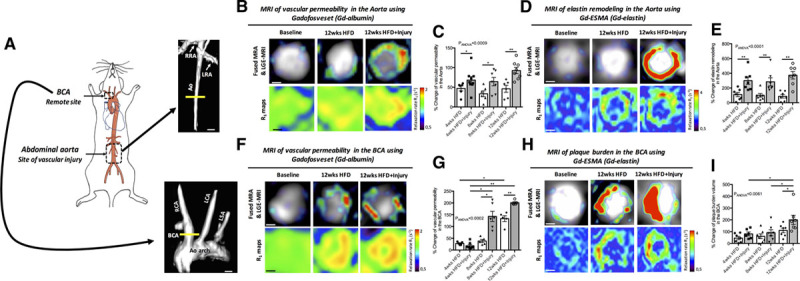
**Sustained arterial inflammation causes a persistent increase plaque permeability and plaque burden in the brachiocephalic artery, remote to the site of injury.** **A**, Schematic of the murine vascular segments evaluated in this study, where vascular injury was performed in the abdominal aorta and the remote arterial segment was the brachiocephalic artery (BCA). **B**, 3D MIP reconstruction shows the level of the abdominal aorta that was imaged. Examples of fused magnetic resonance angiography (MRA) and late gadolinium (Gd) enhancement (LGE)-magnetic resonance imaging (MRI) images of the aorta (**upper** row) and R1 maps (**lower** row) after administration of Gd-albumin, at 12 wk. **C**, Quantification of the percentage change of vascular permeability measured by the relaxation rate (R_1_), after administration of Gd-albumin, at 4, 8, and 12 wk (n=7/group). **D**, Fused MRA and LGE-MRI images of the aorta (**upper** row) and R1 maps (**lower** row) after administration of Gd-elastin at 12 wk. **E**, Quantification of the percentage change in elastin remodeling measured in the LGE-MRI images (n=7/group). **F**, 3D reconstruction of the remote arterial segment imaged shows the brachiocephalic artery (BCA), aortic arch and carotids. Fused MRA and LGE-MRI images (**upper** row) and R1 maps (**lower** row) of the BCA after administration of Gd-albumin at the 12 wk. **G**, Quantification of the percentage change of vascular permeability after administration of Gd-albumin measured by the relaxation rate (R_1_) at 4, 8, and 12 wk (n=7/group). **H**, Fused MRA and LGE-MRI images (**upper** row) and R1 maps (**lower** row) of the BCA after administration of Gd-elastin, at 12 wk. Data were represented as mean±SEM. For multiple-group comparisons, data were analyzed with a Kruskal-Wallis ANOVA with Dunn post hoc test. Ao indicates aorta; HFD, high-fat diet; LCA, left carotid artery; LRA, left renal artery; LSA, left subclavian artery; RCA, right carotid artery; and RRA, right renal artery. *P<0.05 and **P<0.01.

We investigated whether focal vascular injury and subsequent vascular inflammation in the abdominal aorta affects atherosclerosis progression in a remote arterial segment in vivo. For this reason, HFD-fed *ApoE*^−/−^ mice undergoing abdominal aortic injury,^17,26^ which triggers persistent aortic inflammation (Figure III in the Data Supplement), were used to investigate atherosclerosis progression in the BCA (Figure 1A). For serial and comprehensive assessment of changes in vascular pathology at different time points following aortic injury, in vivo MRI with albumin (Gd-albumin) and elastin (Gd-elastin) binding contrast agents that have been validated to measure endothelial permeability^26,30–33^ and plaque burden and remodeling,^34,35^ respectively, were performed (Figure II in the Data Supplement). We found higher vascular permeability (Figure 1B and 1C) and elastin remodeling (Figure 1D and 1E) in the aorta of mice subjected to aortic injury compared with uninjured mice as observed by MRI. In addition, we found that in animals with aortic injury, plaque permeability (Figure 1F and 1G), and plaque burden (Figure 1H and 1I) were consistently increased in the BCA compared with uninjured animals, suggesting that the presence of a focal vascular insult accelerates the progression of atherosclerosis in a remote arterial segment. While we found that plaque permeability in the BCA was significantly higher from 8 weeks onwards following injury compared with uninjured mice (Figure 1F and 1G), plaque burden was significantly higher at 12 weeks, suggesting that changes in plaque permeability precede changes in plaque burden (Figure 1H and 1I). This may suggest a different plaque progression profile in different arterial segments. Functional measurements of endothelial-dependent vasodilation, in response to acetylcholine, showed endothelial dysfunction and paradoxical vasoconstriction^30,36^ in both the injured and uninjured HFD-fed mice at 12 weeks compared with control mice fed a normal laboratory diet as quantified by MRI (Figure IV in the Data Supplement).

### Persistent Vascular Inflammation Promotes Formation of Plaques That Are More Inflamed, Lipid-Rich, and Collagen-Poor in a Remote Arterial Segment

**Figure 2.**
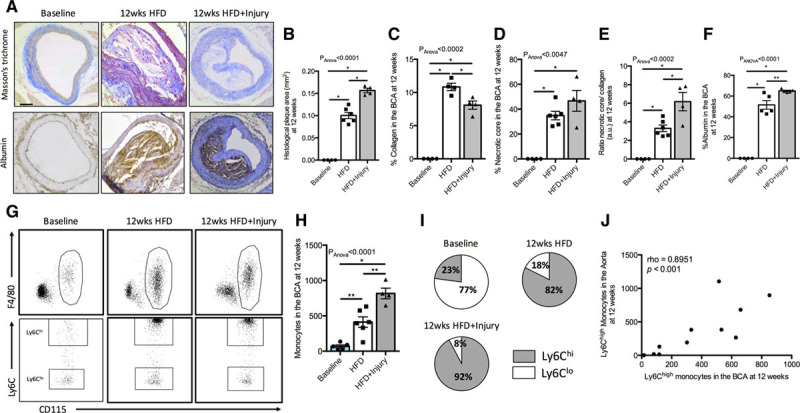
**Persistent arterial inflammation promotes formation of more inflamed, lipid-rich, and collagen-poor plaques in a remote arterial segment.** **A**, Representative trichrome (**upper** row) and albumin immunohistochemistry (**lower** row) stainings of the different experimental groups. Quantification of the (**B**) plaque area, (**C**) collagen content, (**D**) necrotic core, (**E**) ratio of necrotic core to collagen content, and (**F**) albumin at 12 wk in all experimental groups (n=4/group). **G**, Representative flow cytometry images of the gating strategy employed to quantify monocytes in the brachiocephalic artery (BCA) and Ly6C^hi^ and Ly6C^lo^ monocyte subsets in all experimental groups at 12 wk. **H**, Flow cytometric quantification of monocytes in the BCA (n=6/group). **I**, Pie charts show the relative proportion of Ly6C^hi^ and Ly6C^lo^ monocytes in all the groups at the 12 wk time point (n=6/group). **J**, Correlation of Ly6C^hi^ monocytes in the aorta with Ly6C^hi^ monocytes in the BCA at the 12 wk time point. Data were represented as mean±SEM. For multiple-group comparisons, data were analyzed with a Kruskal-Wallis ANOVA with Dunn post hoc test. Correlation data were analyzed with a 2-tailed nonparametric Spearman test. HFD indicates high-fat diet. *P<0.05 and **P<0.01.

To investigate whether arterial inflammation affects atherosclerotic plaque phenotype in remote arteries, we next assessed the compositional characteristics of plaques ex vivo (Figure 2A). We found that BCA atheroma of injured mice had distinct histological features including increased plaque area (Figure 2B), reduced collagen content (Figure 2C), and increased endothelial permeability (% albumin; Figure 2F), compared with uninjured mice. No change in the necrotic core area was found between the 2 groups (Figure 2D). However, the ratio of the necrotic core to collagen was higher in injured mice compared with uninjured mice (Figure 2E). We also found that monocyte infiltration was higher in the BCA of injured compared with uninjured mice (Figure 2G and 2H) whereas no differences in the ratio of tissue Ly6C^hi^ and Ly6C^lo^ monocyte subsets were detected between groups (Figure 2I). Importantly, we found that the amount of Ly6C^hi^ monocytes infiltrating the aortic wall, at the site of injury, correlated with the amount of Ly6C^hi^ infiltration in the remote BCA at 12 weeks. This result suggests that the extent of monocyte-related vascular inflammation, as a result of focal aortic injury, correlates with the monocyte inflammatory burden in the remote artery at 12 weeks (Figure 2J). There were no significant differences between neutrophils, B cells, and T-cell count in the BCA between the 2 groups at 12 weeks (Figure V in the Data Supplement).

**Figure 3.**
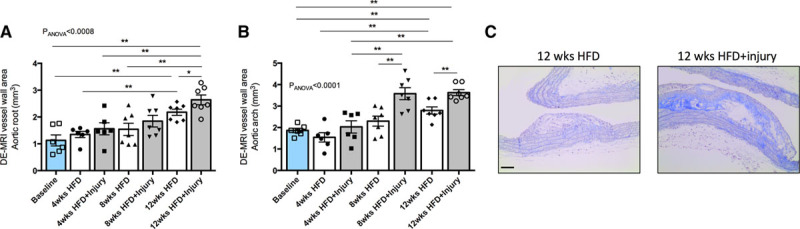
**Aortic injury together with hyperlipidemia promotes plaque progression in the aortic root and aortic arch.** **A**, Quantification of plaque burden in the aortic root and (**B**) aortic arch as measured by the late gadolinium enhancement (LGE)-magnetic resonance imaging (MRI) images (n=6–7/group). **C**, Representative trichrome staining of plaques located in the aortic arch from the different experimental groups. Data were represented as mean±SEM. For multiple-group comparisons, data were analyzed with a Kruskal-Wallis ANOVA with Dunn post hoc test. DE indicates delayed enhancement; and HFD, high-fat diet. *P<0.05 and **P<0.01.

Finally, to evaluate the impact of sustained aortic inflammation on plaque progression in other remote arterial segments, we measured plaque burden in the aortic root and the aortic arch. Similar to the changes observed in the BCA, plaque burden in the aortic root was higher in injured animals compared with noninjured mice at 12 weeks (Figure 3A). Interestingly, differences in plaque burden located in the aortic arch were observed earlier. Injured animals had higher plaque burden in the aortic arch starting from 8 weeks onwards compared with noninjured animals (Figure 3B and 3C).

### Sustained Focal Arterial Inflammation Causes a Persistent Increase of the Circulating Inflammatory Cytokine IL-6

**Figure 4.**
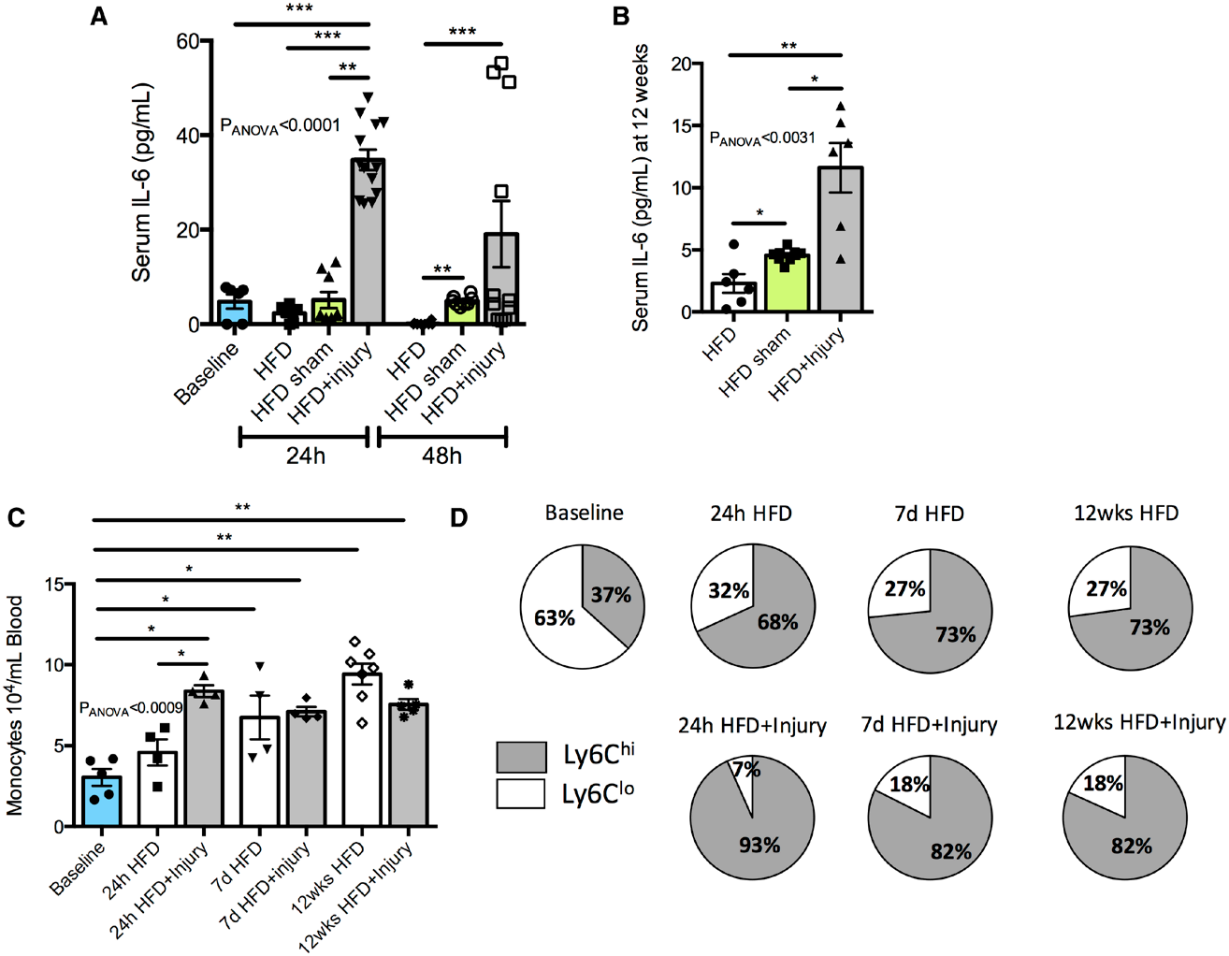
**Sustained arterial inflammation triggers a persistent increase of the inflammatory cytokine interleukin IL (interleukin)-6.** **A**, Quantification of the proinflammatory cytokine IL-6 at the acute phase and (**B**) 12 wk after aortic injury (n=6–9/group). **C**, Flow cytometric quantification of blood monocytes (n=4–6/group). **D**, Pie charts show the relative proportion of Ly6C^hi^ and Ly6C^lo^ monocytes in all the groups at different time points (n=4–6/group). Data were represented as mean±SEM. For multiple-group comparisons, data were analyzed with a Kruskal-Wallis ANOVA with Dunn post hoc test. HFD indicates high-fat diet; hi, high; and lo, low. *P<0.05, **P<0.01, and ***P<0.001.

To investigate whether focal aortic injury affects the inflammatory response at a systemic level, we measured the expression of inflammatory cytokines in serum. We found that IL-6 (interleukin 6) was increased 7-fold in the acute phase (24–48 hours) following aortic injury (Figure 4A) and remained significantly elevated (2.5-fold) up to 12 weeks (Figure 4B). The serum inflammatory markers IL-1β, GM-CSF (granulocyte-macrophage colony-stimulating factor), IL-5, CCL-2 (C-C motif chemokine ligand 2), TNFα (tumor necrosis factor alpha), INFγ (interferon gamma), and VEGF (vascular endothelial growth factor) were below detectability in this murine model. We next evaluated blood monocytes in the acute phase (24 hours and 7 days) and at 12 weeks following injury. A significant increase in circulating monocytes was detected 24 hours after focal aortic injury compared with uninjured mice. However, at later time points no differences were detected between the 2 groups (Figure 4C), both demonstrating monocytosis and promotion of the Ly6C^hi^ subset due to HFD that has previously been reported.^37^ Despite the increased monocyte-related vascular infiltration, at both the site of injury in the abdominal aorta (Figure III in the Data Supplement) and in the remote BCA (Figure 2H), blood monocytes and the percentage of monocyte subsets were similar between injured and uninjured mice (Figure 4C and 4D). The number of circulating neutrophils, B cells, and T cells was similar between the 2 groups at 12 weeks (Figure VI in the Data Supplement).

### Pravastatin and Minocycline Treatments Mitigate the Effects of Sustained Aortic Inflammation on the Progression of Remote Atheroma

**Figure 5.**
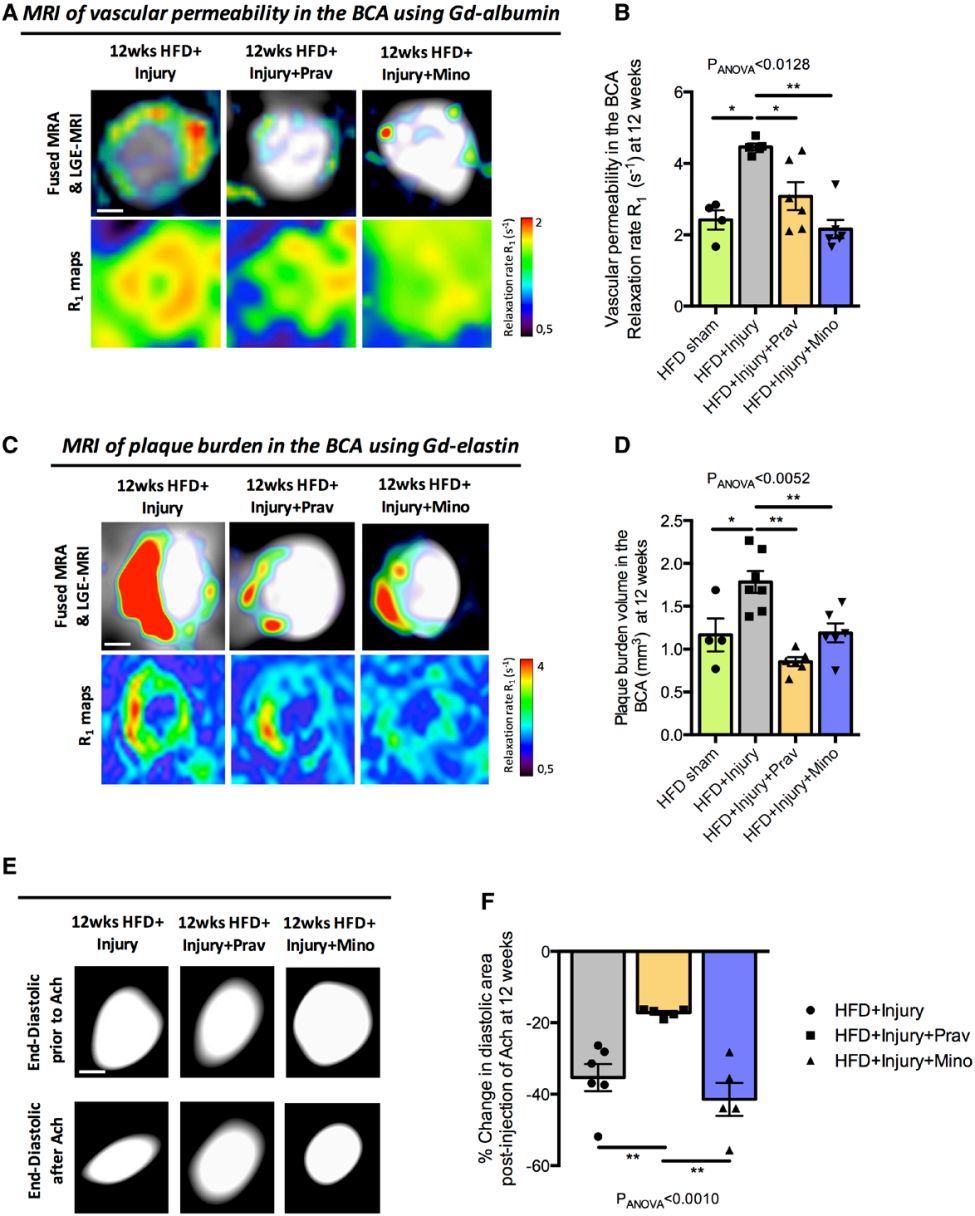
**Oral administration of pravastatin and minocycline decreases plaque permeability and plaque burden in a remote arterial segment.** **A**, Fused magnetic resonance angiography (MRA) and late gadolinium (Gd) enhancement (LGE)-magnetic resonance imaging (MRI) images (**upper** row) and R1 maps (**lower** row) of the brachiocephalic artery (BCA) after administration of Gd-albumin at 12 wk. **B**, Quantification of the plaque permeability after administration of Gd-albumin measured by the relaxation rate (R_1_) at 12 wk (n=6/group). **C**, Fused MRA and LGE-MRI images (**upper** row) and R1 maps (**lower** row) of the BCA after administration of Gd-elastin at 12 wk. **D**, Quantification of plaque burden in the BCA as measured by the LGE-MRI images (n=6/group). **E**, End-diastolic images of the BCA before and after intraperitoneal administration of acetylcholine (Ach). **F**, Quantification of percentage change in diastolic area after Ach administration (n=4–6/group). Data were represented as mean±SEM. For multiple-group comparisons, data were analyzed with a Kruskal-Wallis ANOVA with Dunn post hoc test. HFD indicates high-fat diet; Mino, minocycline; and Prav, pravastatin. *P<0.05 and **P<0.01.

We next investigated if pravastatin, an established therapeutic for atherosclerosis-related diseases and minocycline, an antibiotic treatment with anti-inflammatory properties, could modulate the effects of vascular inflammation, initiated by aortic injury, on plaque progression in the remote artery. To this end, injured mice were treated with either pravastatin or minocycline (Figure II in the Data Supplement) following aortic injury. In vivo MRI at 12 weeks showed that both treatments mitigated the effects of arterial inflammation in the remote artery as demonstrated by reductions in plaque permeability (Figure 5A and 5B) and plaque burden (Figure 5C and 5D) compared with the untreated group. Similar results were obtained when the plaque burden in the aortic root and the aortic arch were analyzed (Figure VII in the Data Supplement). However, only pravastatin treatment improved endothelial-dependent vasodilation, in response to acetylcholine, in the remote artery compared with untreated mice (Figure 5E and 5F).

**Figure 6.**
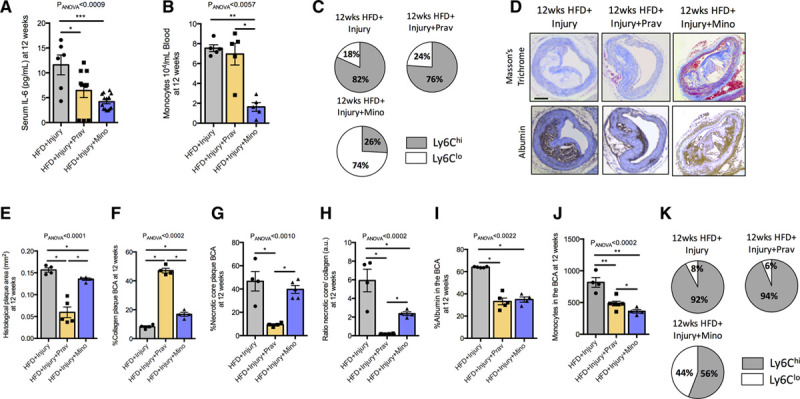
**Pravastatin and minocycline have distinct effects on established drivers of atherosclerosis.** **A**, Quantification of the proinflammatory cytokine IL (interleukin)-6 in serum at 12 wk after aortic injury (n=6–9/group). **B**, Quantification of blood monocytes by flow cytometry (n=6/group). **C**, Pie charts show the relative proportion of Ly6C^hi^ and Ly6C^lo^ blood monocytes in treated and untreated mice at 12 wk (n=6/group). **D**, Representative histological trichrome (**upper** row) and albumin immunohistochemistry (**lower** row) images of the different groups. Quantification of the (**E**) histological plaque area, (**F**) collagen content, (**G**) necrotic core, (**H**) ratio necrotic core to collagen content, and (**I**) albumin at 12 wk in treated and untreated mice (n=4/group). **J**, Flow cytometric quantification of monocytes in the brachiocephalic artery (BCA; n=6/group). Data were represented as mean±SEM. For multiple-group comparisons, data were analyzed with a Kruskal-Wallis ANOVA with Dunn post hoc test. **K**, Pie charts show the relative proportion of Ly6C^hi^ and Ly6C^lo^ monocytes in treated and untreated mice at 12 wk (n=6/group). HFD indicates high-fat diet; hi, high; lo, low; Prav, pravastatin; and Mino, minocycline. *P<0.05, **P<0.01, and ***P<0.001.

To explore the mechanisms by which pravastatin and minocycline treatment impact plaque progression and phenotype in this model, we quantified serum IL-6, blood monocytes, and plaque morphology in the BCA. Pravastatin treatment decreased serum IL-6 (Figure 6A) but did not affect systemic blood monocyte numbers (Figure 6B) and subsets (Figure 6C). However, pravastatin treatment had marked lesion-level effects with significantly reduced plaque size (Figure 6D and 6E), necrotic core size (Figure 6G), necrotic core to collagen ratio (Figure 6H), plaque permeability (Figure 6I), and plaque monocyte infiltration (Figure 6J) compared with untreated mice. Concomitantly, pravastatin treatment significantly increased collagen content (Figure 6F), an established index of plaque stabilization.

Similar to pravastatin, minocycline treatment decreased serum IL-6 (Figure 6A). However, minocycline treatment also reduced monocytes (Figure 6B) and increased the Ly6C^lo^ subset in blood (Figure 6C). Despite the additional impact of minocycline on circulating monocyte numbers and phenotype, minocycline had a smaller impact on remote plaque morphology compared with pravastatin, although minocycline treatment still reduced plaque size (Figure 6D and 6E), necrotic core to collagen ratio (Figure 6H), plaque permeability (Figure 6I), and plaque monocyte infiltration (Figure 6J) with a small increase in collagen content (Figure 6F) compared with untreated mice. No effect was detected on necrotic core size (Figure 6H). Only minocycline treatment resulted in higher numbers of the Ly6C^lo^ monocytes (Figure 6K) within BCA plaque. Overall, these results suggest that both treatments have a beneficial effect in halting plaque progression but likely through different biological pathways.

## Discussion

**Figure 7.**
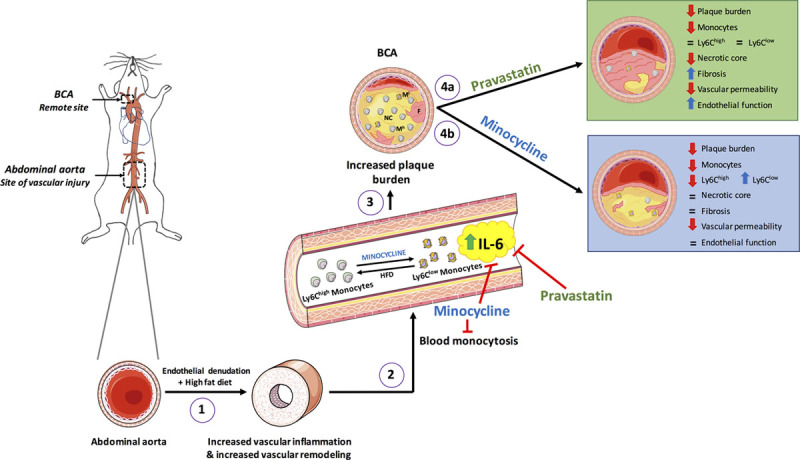
**Take home figure.** Aortic injury in the abdominal aorta together with hyperlipidemic diet promotes vascular inflammation and vascular remodeling at the site of injury. This elicits a systemic inflammatory response characterized by persistently elevated serum IL (interleukin)-6 and acute blood-monocytosis. Blood monocytes infiltrate the brachiocephalic artery (BCA), remote to the site of injury, promoting plaques more inflamed, lipid-rich and collagen-poor compared with uninjured mice. Both pravastatin and minocycline treatments significantly reduce plaque burden but have distinct effects on established mechanistic drivers of atherosclerosis. Pravastatin improved endothelial function, decreased monocyte infiltration and increased collagen content at the BCA in addition to reducing circulating levels of IL-6. However, pravastatin had no significant effect on circulating monocytes. In contrast, minocycline reduced the number of circulating monocytes and promoted a shift to the Ly6C^lo^ vs the Ly6C^hi^ phenotype, in addition to reducing circulating IL-6 levels, with a significant reduction in BCA atheroma volume. HFD indicates high-fat diet.

There is growing evidence to support a central role of systemic inflammation in atherosclerosis progression, with ischemic events exacerbating vascular inflammation-promoting faster plaque growth at a distance. However, whether sustained arterial inflammation is sufficient to cause progression of remote atheroma and modulate plaque characteristics has not been investigated. Using a novel mouse model, this study was the first to reveal the vascular-to-vascular effects of sustained arterial inflammation in the abdominal aorta on the progression and composition of atheroma in a remote arterial segment, the BCA in mice (Figure 7). Molecular and functional MRI, flow cytometry, histology, and Luminex assays revealed that (1) sustained aortic inflammation resulted in the formation of larger and more permeable remote atheroma with higher inflammatory cell count, increased necrotic core, and reduced collagen content; (2) sustained aortic inflammation, triggered by focal aortic injury, has systemic ripple effects triggering more advanced remote atheroma possibly via persistent increased levels of serum IL-6 and increased blood inflammatory monocytes; (3) 2 therapeutic interventions, pravastatin and minocycline, have differential effects on the remote atheroma. Both treatments decreased plaque burden, permeability, and monocyte component and increased collagen in the remote atheroma and alters systemic inflammatory markers including IL-6 and blood monocytes, although to different degrees.

To test the hypothesis that sustained aortic inflammation, in response to focal vascular injury, modulates distant atherosclerotic disease, we serially imaged mice using MRI. Our experimental design enabled noninvasive and direct monitoring of disease progression and response to treatment in multiple arterial segments in vivo. Using molecular and functional MRI, we found that sustained aortic inflammation increased both the permeability and size of the remote atheroma. Interestingly, endothelial function at the site of the remote atheroma was equally decreased in HFD-fed and HFD plus injury mice, suggesting that the additional vascular effect of focal injury did not further impair remote endothelial function. Plaque permeability and endothelial dysfunction are important for initiation and progression of atherosclerosis^38^ and precede plaque rupture and development of cardiovascular events.^38–41^ We and others have demonstrated the utility of molecular MRI to assess atherosclerotic plaque burden.^34,35,42–44^ Importantly, our longitudinal MRI experimental design and the use of 2 contrast agents that measure different biological features of the plaque showed that increased vascular permeability precedes plaque growth; providing further in vivo mechanistic insights about the process of disease progression that was previously only shown in a limited number of studies.^45–48^ Finally, molecular MRI allowed for monitoring the therapeutic effects of pravastatin and minocycline. Both treatments decreased plaque permeability and size of the remote atheroma, as previously reported.^30,34^ However, only pravastatin improved endothelial function, as previously reported.^30^

Detailed ex vivo characterization of the BCA atheroma revealed that sustained aortic inflammation, not only increased remote plaque permeability and size, but also resulted in increased necrotic core to collagen ratio, increased inflammatory monocyte subsets, and reduced plaque collagen. These results are consistent with previous preclinical studies where a preexisting underlying proinflammatory cardiovascular insult, for example, acute myocardial infarction and necrosis, leads to accelerated atherosclerosis due to sympathetically driven systemic inflammation^3^ and upregulation of inflammatory adhesion molecules on the plaque surface.^4^ In line with our work, clinical studies have demonstrated the association between plaques and their complexities in different vascular segments in patients with and without myocardial infarction^49^ and the possible role of local and systemic inflammation on plaque instability.^11,15^ However, the propagation of vascular inflammation, downstream impact on atheroma progression, and mechanisms underlying this interaction remain obscure.

To assess possible mechanisms by which aortic injury affects the composition of remote atheroma, we investigated the systemic inflammatory response. Unlike in myocardial infarction models, our model showed persistent vascular inflammation up to 12 weeks following aortic injury, characterized by increased monocyte infiltration at the site of injury and at the remote atheroma with an associated increase in systemic inflammatory blood monocytes and serum IL-6, that could contribute to both, the development of focal vascular remodeling and remote atherosclerosis progression. Several studies have shown that IL-6 exerts a broad range of biological roles by promoting a proinflammatory response and regulating cholesterol homeostasis.^38,50^ Moreover, serum IL-6 levels have been shown to strongly associate with atherothrombosis and future vascular events, independent of traditional risk factors in patients with coronary heart disease.^51^ Modulation of systemic IL-6 by Canakinumab reduced or prevented atherosclerosis-related cardiovascular events, as shown in the CANTOS study.^15,16^ Importantly, several studies have demonstrated an impact of IL-6 on atherosclerosis via numerous mechanisms including chemotactic of monocytes and activation of endothelial cells with subsequent production of chemokines and adhesion molecules,^50^ which correlates with fatty streak formation.^52^ However, other studies have shown that knock-down of IL-6 in ApoE^−/−^ mice, exacerbates atherosclerosis and decreases leukocyte homing,^53,54^ which could be related to the IL-6-dependent regulation of lipid metabolism. In this context, IL-6 can act in an atheroprotective manner by increasing cholesterol efflux to apoA1 (apolipoprotein A1) in macrophages by upregulating ABC (ATP-binding cassette transporter) A1.^55^ In addition, high IL-6 levels have been associated with low HDL (high-density lipoproteins), which is involved in cholesterol transport back to the circulating blood, anti-thrombotic actions, dampening inflammation, and lowering oxidative stress.^56,57^ Therefore, we speculate that sustained increased in serum IL-6 observed in the injured animals in our study contributes to the propagation of vascular inflammation and infiltration of monocytes at the site of injury and subsequently in the remote atheroma. However, additional experiments, for example, using ApoE^−/−^/IL-6 animals or downregulation of the IL-6 using miRNAs or antibodies are needed to elucidate which cells produce IL-6, and whether the effects of IL-6 are causal.

Finally, we evaluated the systemic and lesion-level effects of 2 pharmacological interventions, pravastatin and minocycline. Our data demonstrated that pravastatin treatment in animals with sustained arterial inflammation improved both anatomic and functional metrics of the remote atheroma. Specifically, pravastatin treatment reduced plaque permeability, burden, necrotic core, and monocyte infiltration; increased collagen and improved endothelial function in the remote atheroma. At the systemic level, pravastatin treatment reduced serum IL-6, but surprisingly had no impact on the number or phenotype of circulating monocytes. It is well established that treatment with statins have unequivocal benefits in reducing the rate of plaque progression and cardiovascular events and changing plaque composition in both, animal models^30,58,59^ and patients with coronary artery disease.^60^ We and others have previously shown the beneficial effects of pravastatin treatment in reducing vascular permeability, plaque burden, and improving endothelial function in both mice^30,34^ and humans.^61^ In addition, a recent study has shown that intravenous administration of atorvastatin during myocardial infarction limits cardiac damage, improves cardiac function, and mitigates remodeling to a larger extent than when administered orally shortly after reperfusion.^62^ In this study, we demonstrate for the first time the beneficial impact of statin treatment in mitigating the vascular-to-vascular effects of sustained aortic inflammation. Our data are also in agreement with studies showing that pravastatin promotes plaque stabilization by increasing the collagen content and reducing inflammation, in a dose-dependent manner, reducing plaque lipids, metalloproteinases, and cellular death in different vascular segments in both patients^60,63^ and animals.^59,64^

Treatment with minocycline, a tetracycline derived antibiotic, reduced plaque permeability, burden, necrotic core to collagen ratio, monocyte infiltration and increased collagen, but did not improve endothelial-dependent vasodilation. Unlike pravastatin, minocycline had a marked effect on both, tissue and systemic inflammation. Minocycline treatment caused a reduction in both circulating and lesion monocyte content with a shift towards more reparative Ly6C^lo^ monocyte subsets, in addition to a decrease serum IL-6 levels. Previous preclinical studies have shown that treatment with minocycline, caused a reduction in plaque progression and promoted stabilization of plaques by inhibiting vascular smooth muscle cell proliferation, reducing plaque MMP (matrix metallopeptidases) activity,^27–29^ and reducing vascular permeability^29^ in models of atherosclerosis. Our finding that minocycline affects monocyte polarization is novel and has not been previously reported. Previous studies only showed that minocycline inhibited microglia polarization to a proinflammatory state in murine models of amyotrophic lateral sclerosis.^65^ Additional experiments are needed to explore the molecular mechanisms by which minocycline modulates monocyte polarization. Our finding that minocycline reduces circulating levels of IL-6 is consistent with previous work showing that minocycline treatment supresses IL-6 expression in ovarian cancer cells^66^ and in the central nervous system, acting as a neuroprotective agent.^67,68^ However, future studies are needed to elucidate the molecular pathways by which minocycline decreases IL-6 expression.

There is a limitation associated with the current study. Quantification of the re-endothelialization process in the injured abdominal aorta at different time points for this animal model has not been performed. CD31 immunohistochemistry or even high-resolution transmission electron microscopy images would have provided important information about this process and will be evaluated in our future studies.

In conclusion, we demonstrate that a single mechanical injury in the aorta results in sustained aortic inflammation and is sufficient to exert remote vascular-to-vascular effects that accelerate remote atheroma formation leading to plaques that are more inflamed, lipid-rich and collagen-poor, in the absence of tissue necrosis or myocardial infarction. This may involve systemic ripple effects of vascular inflammation via elevated levels of circulating IL-6 and inflammatory blood monocytes. The vascular-to-vascular effects can be inhibited by pravastatin or minocycline treatments, which reduce IL-6 but have distinct effects on circulating monocytes. These results provide possible mechanistic insights into the higher incidence of remote atherothrombotic complications following an index cardiovascular event and of potential therapeutic targets to mitigate disease progression.

## Acknowledgments

We would like to thank Prakash Saha, Richard Beatson and Sean O’Farrell for providing experimental advice on different aspects of this work and David Onthank (Lantheus Medical Imaging) for providing Gd-ESMA.

## Sources of Funding

This work was supported by the following grants: (1) Engineering and Physical Sciences Research Council (EPSRC) EP/P032311/1, EP/P001009/1 and EP/P007619/1, (2) British Heart Foundation (BHF) programme grant RG/20/1/34802, (3) King’s BHF Centre for Research Excellence RE/18/2/34213 (4) Wellcome EPSRC Centre for Medical Engineering (NS/A000049/1), (5) FONDECYT 1180525, and (6) the Department of Health via the National Institute for Health Research (NIHR) Cardiovascular Health Technology Cooperative (HTC) and comprehensive Biomedical Research Centre awarded to Guy’s & St Thomas’ NHS Foundation Trust in partnership with King’s College London and King’s College Hospital National Health Service Foundation Trust.

## Disclosures

None.

## Supplementary Material


