# Supplementary figures and images for: Sustained Focal Vascular Inflammation Accelerates Atherosclerosis in Remote Arteries
Source: Arterioscler Thromb Vasc Biol. 2020 Jul 16;40(9):2159–70. doi: 10.1161/ATVBAHA.120.314387 (PMC7447189; doi:10.1161/ATVBAHA.120.314387)

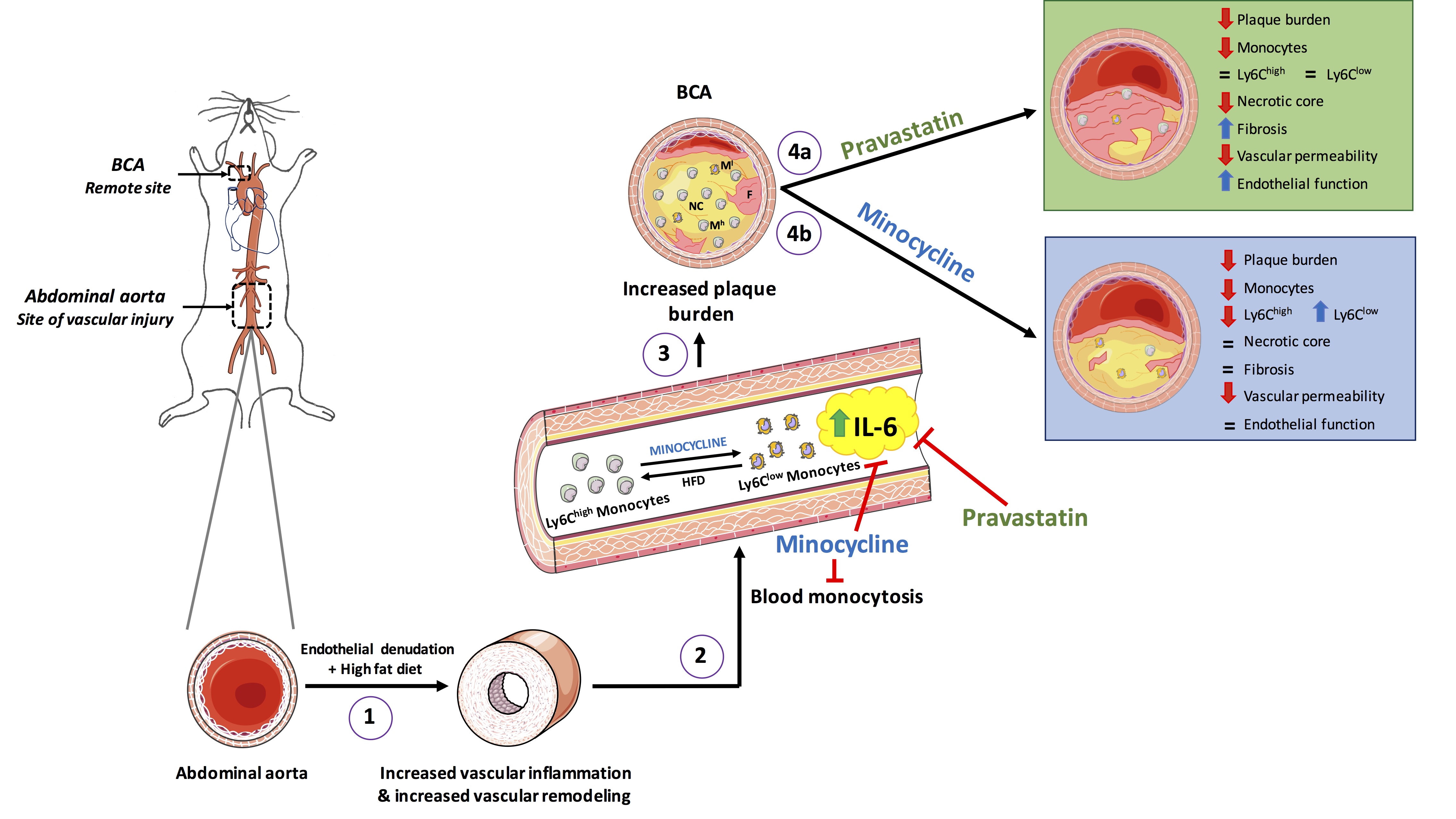

Supplement: Supplementary file 1 [file atv-40-2159-s001.jpg]
