# Supplementary material for: Sustained Focal Vascular Inflammation Accelerates Atherosclerosis in Remote Arteries
Source: Arterioscler Thromb Vasc Biol. 2020 Jul 16;40(9):2159–70. doi: 10.1161/ATVBAHA.120.314387 (PMC7447189; doi:10.1161/ATVBAHA.120.314387)
Supplement: Supplementary file 2 [file atv-40-2159-s002.pdf]

# SUPPLEMENTAL MATERIALS

## SUSTAINED FOCAL VASCULAR INFLAMMATION ACCELERATES ATHEROSCLEROSIS IN REMOTE ARTERIES

**Begoña Lavin[1*] PhD; Alkystis Phinikaridou[1] PhD; Marcelo E. Andia[2] MD, PhD; Myles Potter[1]; Silvia Lorrio[1] PhD; Imran Rashid[1,3] MD, PhD; Rene M. Botnar[1,4] PhD.**

[1]School of Biomedical Engineering and Imaging Sciences, King's College London, UK.

[2]Radiology Department & Millennium Nucleus for Cardiovascular Magnetic Resonance, Pontificia Universidad Católica de Chile.

[3]Case Cardiovascular Research Institute, Case Western Reserve University, Cleveland, Ohio 44106, USA.

[4]Escuela de Ingeniería, Pontificia Universidad Católica de Chile, Santiago, Chile.

**Aortic injury of the abdominal aorta**

Surgery was performed under isoflurane anaesthesia using a dissection microscope (Leica, Wetzar, Germany). Prior to surgery, fur was removed, and the surgical field was disinfected. After a midline incision of the abdomen, the aorta, renal, and iliac arteries were exposed and isolated. Subsequently, two ligatures (surgical silk, size 4-0, Aragó, Zaragoza, Spain) were placed around the aorta, below the renal arteries and above the iliac bifurcation, respectively. A 30G syringe was introduced into the abdominal aorta followed by a total infusion of 1mL saline buffer, that was administered in 3 boluses of 300-350L each, to remove the endothelial cells (Supplemental Figure I). Finally, the aortic puncture was repaired, the ligatures were removed, the muscle and skin were sutured (5-0 Vicryl, Ethicon Inc., Somerville, NJ, USA) and the animals were allowed to recover. Each surgery lasted 30min. Sham-operated animals underwent the same surgical procedure but without injuring the endothelial layer.

***In vivo* MRI protocol**

*In vivo* MRI of the arterial vessel wall was performed using a Philips Achieva MR scanner (Philips Healthcare, Best, The Netherlands) equipped with a clinical gradient system (30 mT $m^{-1}$, 200 mT $m^{-1}$ $ms^{-1}$). All animals were scanned twice. For the first imaging session, mice were scanned 30min after intravenous administration of gadofosveset (Gd-albumin, 0.03mmol/kg). After a washout period of 24h, mice underwent a second imaging session 1h after intravenous administration of an elastin-specific gadolinium-based MR contrast agent (Gd-elastin; 0.2mmol/kg). Both contrast agents were obtained from Lantheus Medical Imaging (North Billerica, MA, USA). In each mouse, two vascular segments were imaged at each time point. First, the abdominal aorta was imaged using a single-loop surface coil (diameter=47mm) and

secondly, the brachiocephalic artery (BCA) was imaged using a single-loop surface coil (diameter=23mm). For imaging the aorta, mice were placed in a supine position while for imaging the brachiocephalic artery (BCA) mice were placed in a prone position. Anaesthesia was induced with 5% and maintained with 1-2% isoflurane mixed in medical oxygen. For visualization of the vasculature contrast-enhanced angiography images were acquired. The maximum intensity projection (MIP) images were used to plan the subsequent late gadolinium enhancement (LGE) and T1 mapping scans. A 2D-Look–Locker sequence was planned perpendicular to the aorta and BCA to determine the optimal inversion time (TI) for blood signal nulling. 3D LGE fast gradient echo sequence was used for visualization of contrast agent uptake. 3D T1 mapping was performed using a Modified-Look-Locker sequence that employs non-selective inversion pulses with inversion times ranging from 20 to 2000ms, followed by eight segmented readouts for eight individual images. The two interleaved imaging trains employed to achieve higher temporal resolution result in a set of 16 images per slice with increasing inversion times. MRI acquisition parameters are summarised in supplemental tables 1 and 2. N=5-9 mice were analysed per group.

**MRI vessel vasomotor response to acetylcholine**

A cohort of $ApoE^{-/-}$ mice in the control, HFD, HFD+injury and HFD+injury+treatments (n=4-6 per group) groups were used to evaluate the endothelial-depended vasomotor response of the BCA after acetylcholine administration at 12 weeks. Transverse 2D gradient echo (GRE) retrospective ECG gated images were acquired prior to and 6min after intraperitoneal acetylcholine administration (16.6mg/kg, Sigma, Dorset, UK). The acquisition parameters were: FOV=40x23mm, in-plane resolution=0.3x0.3x1mm, matrix=132x77, TR/TE=9.4/4.5ms, flip angle=30º, cardiac phases=14, scan time≈2min.

**Magnetic resonance imaging analysis**

Analysis was performed using OsiriX (OsiriX Foundation, Geneva, Switzerland). To register the lumen and vessel wall of the aorta and BCA, angiography images were co-registered and overlaid with high-resolution LGE-MRI images. Vascular permeability and elastin remodeling in the abdominal aorta and BCA were measured by manually segmenting the LGE-MRI images and T1 maps after administration of Gd-albumin or Gd-ESMA, respectively on consecutive slices along the vessel. Relaxation rate (R1=1/T1, $s^{-1}$) values obtained from consecutive T1 mapping slices were averaged for each animal. LGE-MRI measurements from consecutive slices were added for each animal and multiply by the slice thickness, to express the total volume of vascular permeability, vessel wall remodeling and plaque burden, respectively. For the longitudinal study (Fig. 1) data were presented as % change of vascular permeability (VP), elastin remodeling (ER) and plaque burden (PB) using the following equation:

$$\%\ Change\ VP = \frac{VP_t - VP_{baseline}}{VP_{baseline}}$$

$$\%\ Change\ ER = \frac{ER_t - ER_{baseline}}{ER_{baseline}}$$

$$\%\ Change\ PB = \frac{PB_t - PB_{baseline}}{PB_{baseline}}$$

t=4, 8 and 12 weeks after commencement of the experimental protocol.

To assess the endothelial-dependent vasodilation response of the BCA, the luminal end-diastolic areas were compared before and 6min after administration of acetylcholine (Ach). Data were presented as the % change of diastolic area (DA) using the following equation:

$$\% \, Change \ of \ DA = \frac{DA_{postAch} - DA_{preAch}}{DA_{preAch}}$$

**Histology**

Following the MRI scans at 12weeks, mice were culled and tissues were collected for *ex vivo* analysis. For all histological procedures mice were anesthetized with isoflurane and perfused through the left ventricle with physiological saline. The aortic root, aortic arch and BCA arteries were removed in block and the abdominal aorta, between the renal branches and the iliac bifurcation was removed separately. The samples were immediately fixed with 10% buffered formalin for 48h at 4$^{o}$C, embedded in paraffin, and sectioned transversely (5µm thick). Masson's trichrome stain (Sigma-Aldrich, Dorset, UK) was used to visualise vessel wall morphology, and quantify collagen content. Immunohistochemistry (IHQ) for serum albumin was performed using and anti-mouse goat monoclonal antibody (1:5000, Abcam) and for macrophages using an anti-mouse rabbit polyclonal antibody against CD68 (1:100, Abcam). Vascular remodeling in the abdominal aorta and plaque burden in the BCA was calculated using the trichrome images [adventitia area - luminal area, ($mm^2$)]. For vessel wall albumin analysis the immunopositive areas were segmented on the images and expressed as percentage area of albumin [(%) = (immunopositive area/ total area)*100]. Histological quantifications were performed using ImageJ software (NIH, Bethesda, MD). Individual measurements performed within each animal were averaged and 4 animals were analysed per group.

**Flow cytometry analysis**

Blood samples were collected by terminal cardiac puncture. The vascular segments (BCA and aorta) were sliced in small rings and incubated in 1mL of dissociation enzyme solution containing 125U/mL Collagenase type XI, 60U/mL Hyaluronidase type 1-s, 60U/mL DNase I, and 450U/mL Collagenase type I in 2.5mL

of PBS for 1h at 37°C. Then, cells were passed through a 70μm cell strainer. Then, all samples were incubated with the corresponding antibodies: CD115 (2,5μg $ml^{-1}$; Thermo Fisher; clone: AFS98), F4/80 (1μg $ml^{-1}$; Thermo Fisher; clone: BM8), Ly-6C (0,5μg $ml^{-1}$; Thermo Fisher; clone: HK1.4), B220 (0,5μg $ml^{-1}$; Thermo Fisher; clone: RA3-6B2), CD90 (0,5μg $ml^{-1}$; Thermo Fisher; clone: eBio5E10), NK1.1 (0,5μg $ml^{-1}$; Thermo Fisher; clone: PK136), Ly6G (0,5μg $ml^{-1}$; Thermo Fisher; clone: 1A8), CD45 (1μg $ml^{-1}$; Thermo Fisher; clone:30-F11), CD19 (1μg $ml^{-1}$; Thermo Fisher; clone: eBio1D3), CD3 (1μg $ml^{-1}$; Thermo Fisher; clone: 145-2C11), Ly6G (2,5μg $ml^{-1}$; Thermo Fisher; clone: 1A8) and CD11b (1μg $ml^{-1}$; Thermo Fisher; clone: M1/70). Subsequently, samples were cleared from red blood cells and platelets using a lysis buffer (FACS lysing solution, BD Bioscience). Finally, samples were fixed in 1% paraformaldehyde. All samples were analysed using a FACSCanto flow cytometer and a FACSDiva software (BD Biosciences) and analysed using FlowJo Version 10 software (Ashland, US). N=4-6 animals were analysed per group.

**Cytokine quantification in serum (Luminex assay)**

Mouse serum samples were assayed for IL-6, IL-5, CCL-2, GM-CSF, IFNγ, IL-1ß, TNFα and VEGF using a Mouse Magnetic Luminex Assay according to the manufacturer instructions (R&D Systems, Minneapolis, US). Blood was collected and allowed to clot for 1h at room temperature before centrifuging for 20min at 2000xg. Then serum was aliquoted and stored at -20°C until was used for the cytokine quantification. 6-9 animals were analysed per group.

**Table major resources**

**Animals (in vivo studies)**

| Species | Vendor or Source | Background Strain | Sex | Persistent ID / URL |
|---|---|---|---|---|
| Mice | Charles River Laboratories | B6.129P2-Apoe$^{tm1Unc}$/J | Male | https://www.criver.com/products-services/find-model/jax-apoe-mice?region=3661 |

**Antibodies**

| Target antigen | Vendor or Source | Catalog # | Working concentration | Persistent ID / URL |
|---|---|---|---|---|
| Serum albumin | Abcam | ab8940 | 0.00394 μg/μL | https://www.abcam.com/human-serum-albumin-antibody-ab8940.html |
| CD68 | Abcam | ab125212 | 5 μg/μL | https://www.abcam.com/cd68-antibody-ab125212.html |
| CD115 | Thermo Fisher | 53-1152-82 | 2.5 μg/μL | https://www.thermofisher.com/antibody/product/CD115-c-fms-Antibody-clone-AFS98-Monoclonal/53-1152-82 |
| F4/80 | Thermo Fisher | 17-4801-80 | 1 μg/μL | https://www.thermofisher.com/antibody/product/F4-80-Antibody-clone-BM8-Monoclonal/17-4801-80 |
| Ly6C | Thermo Fisher | 12-5932-82 | 0.5 μg/μL | https://www.thermofisher.com/antibody/product/Ly-6C-Antibody-clone-HK1-4-Monoclonal/12-5932-82 |
| B220 | Thermo Fisher | 45-0452-82 | 0.5 μg/μL | https://www.thermofisher.com/antibody/product/CD45R-B220-Antibody-Monoclonal/45-0452-82 |
| CD90 | Thermo Fisher | 45-0909-42 | 0.5 μg/μL | https://www.thermofisher.com/antibody/product/CD90-Thy-1-Antibody-clone-eBio5E10-5E10-Monoclonal/45-0909-42 |
| NK1.1 | Thermo Fisher | 45-5941-82 | 0.5 μg/μL | https://www.thermofisher.com/antibody/product/NK1-1-Antibody-Monoclonal/45-5941-82 |
| Ly6G | Thermo Fisher | 11-9668-82 | 0.5 μg/μL | https://www.thermofisher.com/antibody/product/Ly-6G-Antibody-Monoclonal/11-9668-82 |
| CD45 | Thermo Fisher | 45-0451-82 | 1 μg/μL | https://www.thermofisher.com/antibody/product/CD45-Antibody-Monoclonal/45-0451-82 |
| CD19 | Thermo Fisher | 12-0193-82 | 1 μg/μL | https://www.thermofisher.com/antibody/product/CD19-Antibody-Monoclonal/12-0193-82 |
| CD3 | Thermo Fisher | 47-0031-82 | 1 μg/μL | https://www.thermofisher.com/antibody/product/CD3e-Antibody-Monoclonal/47-0031-82 |
| Ly6G | Thermo Fisher | 46-9668-82 | 2.5 μg/μL | https://www.thermofisher.com/antibody/product/Ly-6G-Antibody-Monoclonal/46-9668-82 |
| CD11b | Thermo Fisher | 12-0112-82 | 1 μg/μL | https://www.thermofisher.com/antibody/product/CD11b-Antibody-Monoclonal/12-0112-82 |

**Others**

| Description | Vendor or Source | Catalog # | Working concentration | Persistent ID / URL |
|---|---|---|---|---|
| Lysing Solution 10X Concentrate | BD Bioscience | 349202 | 1x | https://www.bdbiosciences.com/eu/applications/clinical/blood-cell-disorders/other-reagents/sample-prep-reagents/lysing-solution-10x-concentrate/p/349202 |
| Collagenase type XI | Sigma Aldrich | C7657-100MG | 125U/mL | https://www.sigmaaldrich.com/catalog/product/sigma/c7657?lang=es&region=ES |
| Hyaluronidase type 1-s | Sigma Aldrich | H3506-100MG | 60U/mL | https://www.sigmaaldrich.com/catalog/product/sigma/h3506?lang=es&region=ES |
| DNase I | Sigma Aldrich | D4263-1VL | 60U/mL | https://www.sigmaaldrich.com/catalog/product/sigma/d4263?lang=es&region=ES |
| Collagenase type I | Sigma Aldrich | SCR103 | 450U/mL | https://www.sigmaaldrich.com/catalog/product/mm/scr103?lang=es&region=ES |
| Mouse Magnetic Luminex Assay | R&D Systems | LXSAMSM | | https://www.rndsystems.com/products/mouse-magnetic-luminex-assay_lxsamsm |
| Gd-albumin (Ablavar) | Lantheus Medical Imaging | | 0.03 mmol/kg | https://www.lantheus.com/ |
| Gd-elastin | Lantheus Medical Imaging | LMI1174 | 0.2 mmol/kg | https://www.lantheus.com/ |
| Acetylcholine | Sigma Aldrich | A6625-100G | 16.6mg/kg | https://www.sigmaaldrich.com/catalog/product/sigma/a6625?lang=en&region=GB |
| Minocycline | Mylan | | 3mg/kg/day | https://www.mylan.com/ |
| Pravastatin | Kemprotec Limited | 81131-70-6 | 40mg/kg/day | https://www.kemprotec.co.uk/search_results.php?_search=Pravastatin%20sodium |

**Statistical analysis**

The sample sizes included in the full study were calculated based on pilot MRI data acquired at 12 weeks in injured and non-inured mice showing that detection of changes in plaque burden between these groups when using MRI with Gd-Albumin

required 3-6 animals per group (133.5 ± 27.70 mm3 vs 201.1 ± 9.317 mm3). When using the Gd-elastin 5-7 animals per group were required (87.79 ± 64.15 mm3 vs 373.3 ± 134.2 mm3). For *ex vivo* analysis, including histology, flow cytometry and Luminex, the sample size was calculated based on the same principles as for the MRI data showing that detection of differences between these groups required 4-6 animals per group (Histology [plaque size]: 0.1003 ± 0.017 mm2 vs 0.1565 ± 0.010 mm3; Flow cytometry [total monocytes]: 411.8 ± 181.4 vs 816.0 ± 150.7; Luminex [IL-6] 4.56 ± 0.49pg/mL vs 11.60 ± 4.88pg/mL).

**Supplemental material**

**Supplemental Figure I**

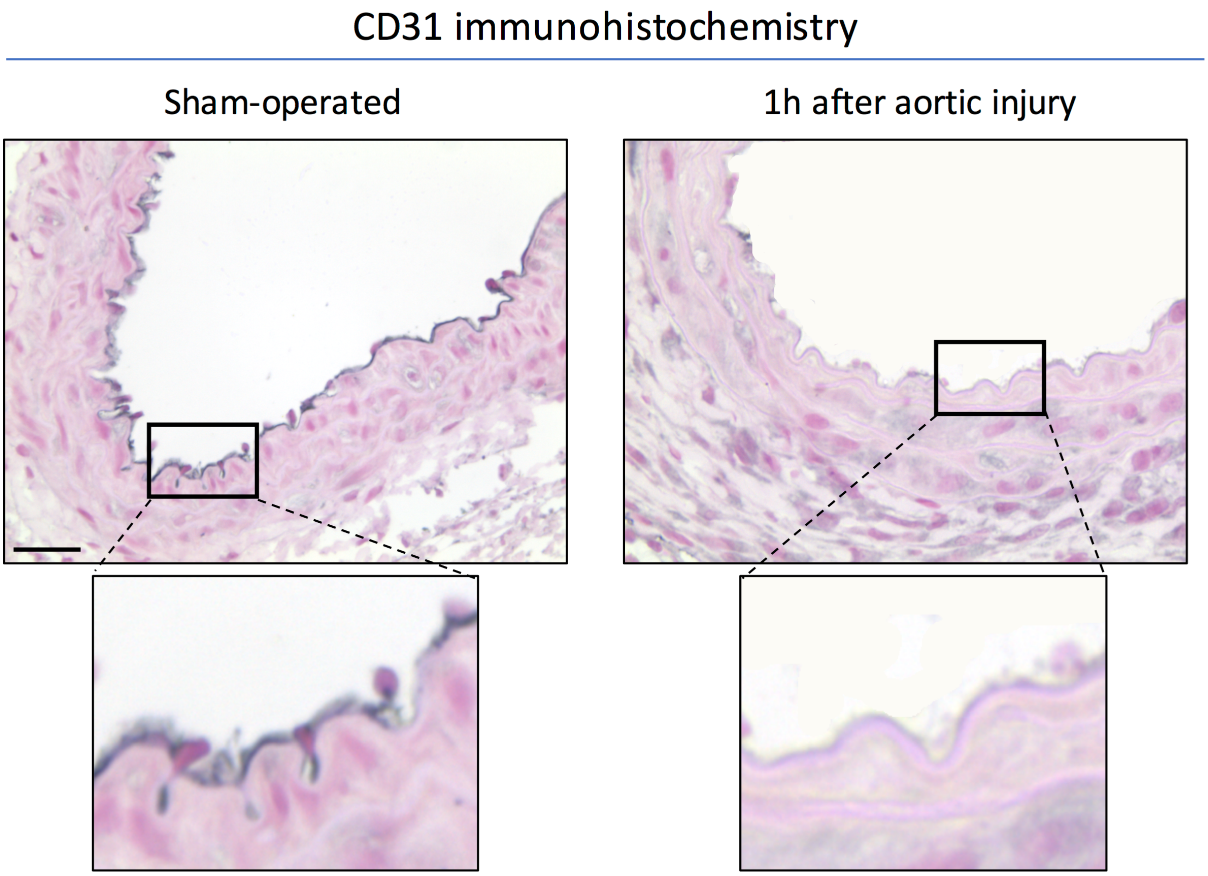


**Supplemental Figure I. Endothelial denudation protocol.** CD31 immunohistochemistry showing the presence of endothelial cells in sham-operated animals (left) and the absence of endothelial cells 1 hour after injury (right).

## Supplemental Figure II

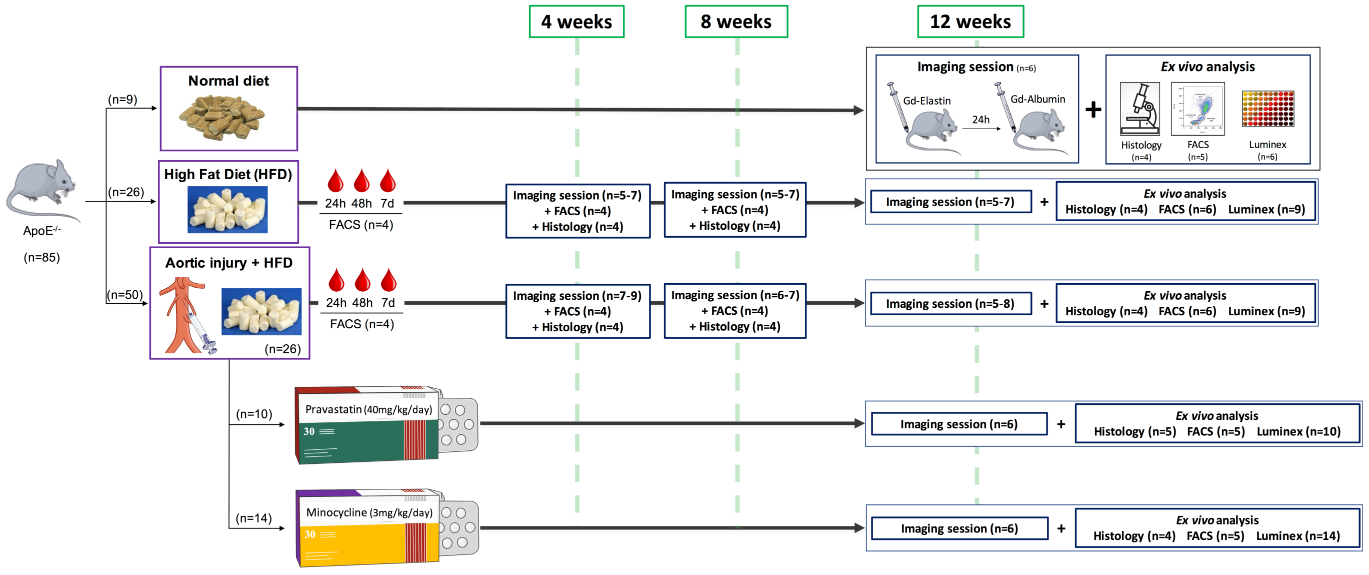


**Supplemental Figure II. Experimental design.** FACS: Flow cytometry; Gd: gadolinium.

## Supplemental Figure III

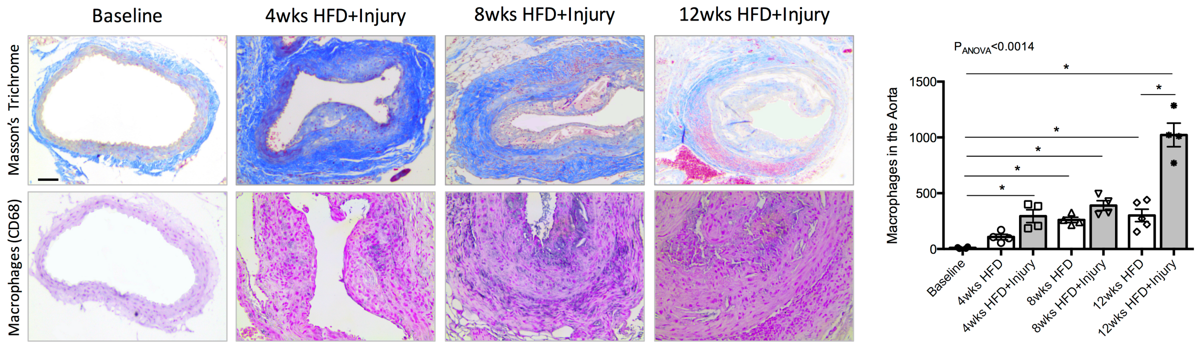


**Supplemental Figure III. Aortic injury together with hyperlipidaemia is a model of persistent vascular inflammation.** Representative trichrome histological images of the abdominal aorta, at the site of injury, prior to and at 4, 8 and 12 weeks after aortic injury (upper row). Immunohistochemistry for CD68 to allow visualization of macrophages infiltrated in the abdominal aorta at the site of injury (lower row) and quantification of total macrophages infiltrated in the aorta at the site of injury measured by flow cytometry as a measurement of persistent vascular inflammation. (n=6/group). Data were represented as mean±sem. For multiple-group comparisons, data were analysed with a Kruskal-Wallis ANOVA with Dunn's post hoc test. HFD: high fat diet. *P<0.05.

**Supplemental Figure IV**

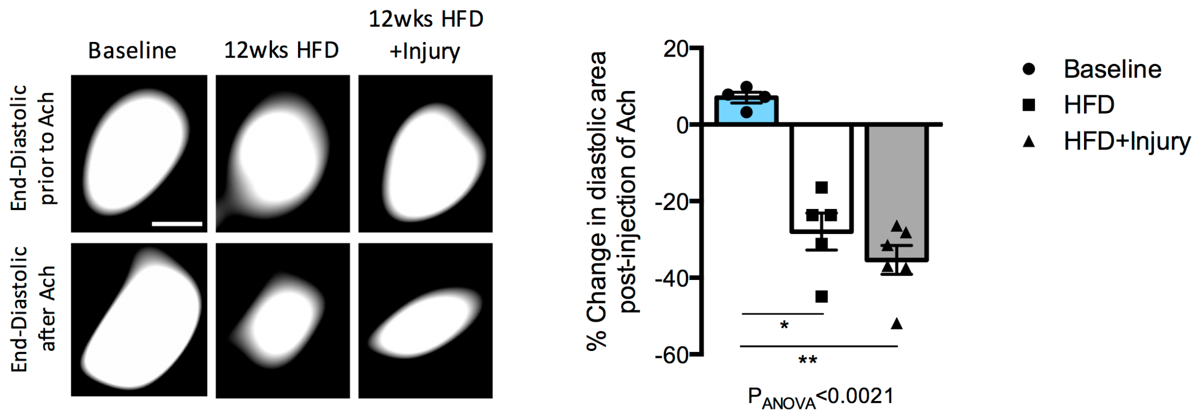


**Supplemental Figure IV. Sustained vascular inflammation, initiated by aortic injury, has not significant effect on the vasodilatory properties in the remote arterial segment compared with mice fed-HFD alone.** Bright blood cardiac triggered MRI images show end-diastolic images of the BCA before and after intravenous administration of acetylcholine showing paradoxical vasoconstriction in mice that were fed HFD, but not significant differences between injured and uninjured mice (n=4-6/group). Data were represented as mean±sem. For multiple-group comparisons, data were analysed with a Kruskal-Wallis ANOVA with Dunn's post hoc test. HFD: high fat diet; BCA: brachiocephalic artery; Ach: acetylcholine. *P<0.05, **P<0.01.

**Supplemental Figure V**

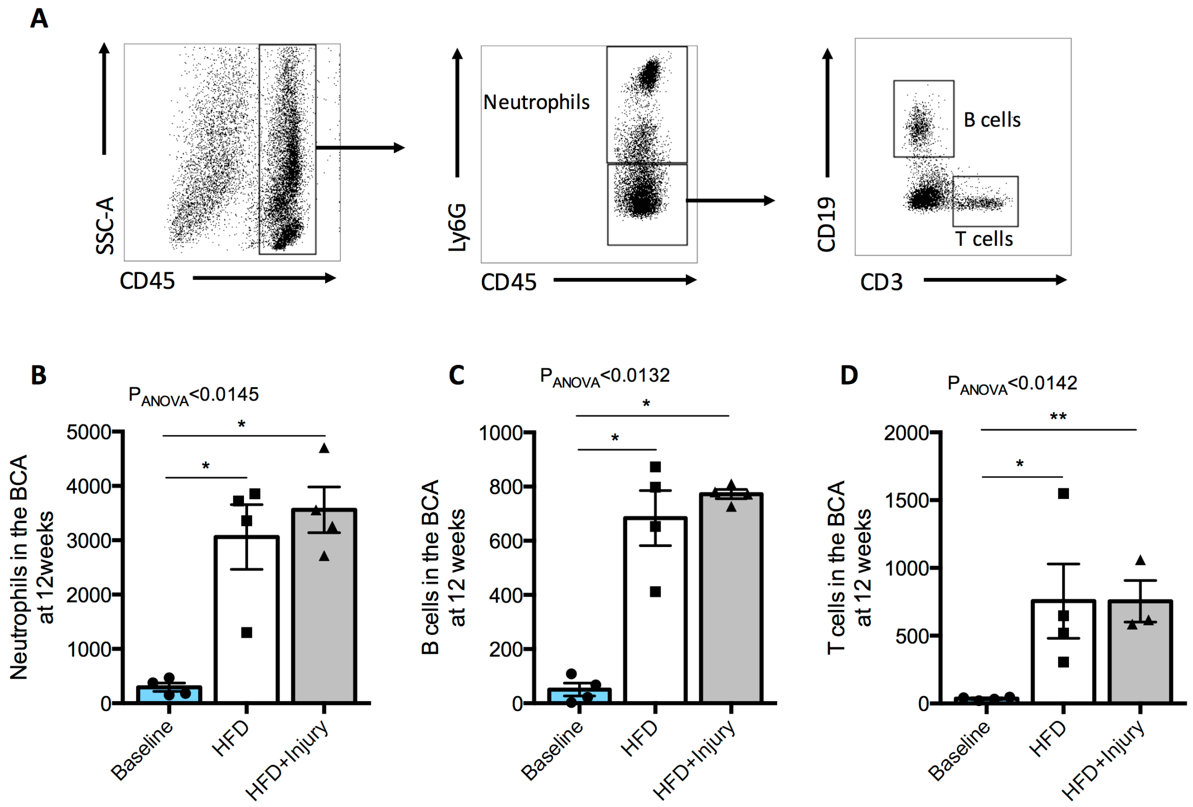


**Supplemental Figure V. Similar infiltration of neutrophils, B and T cells was measured in the BCA between injured and uninjured mice at 12 weeks. A,** Flow cytometry gating strategy employed to quantify neutrophils, B cells and T cells in the BCA. Flow cytometric quantification of **(B)** neutrophils, **(C)** B cells and **(D)** T cells in the BCA at 12 weeks (n=6/group). Data were represented as mean±sem. For multiple-group comparisons, data were analysed with a Kruskal-Wallis ANOVA with Dunn's post hoc test. BCA: brachiocephalic artery; HFD: High fat diet. *P<0.05, **P<0.01.

**Supplemental Figure VI**

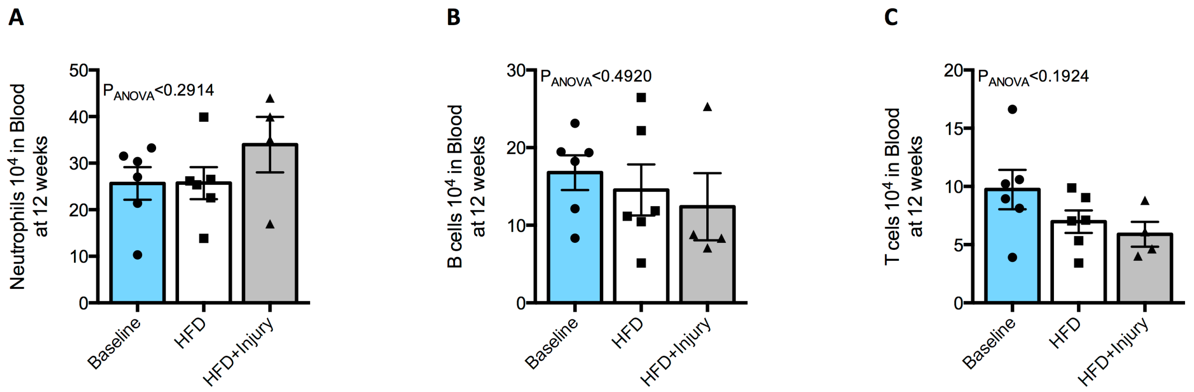


**Supplemental Figure VI. Similar content of neutrophils, B and T cells were measured in blood between injured and uninjured mice at 12 weeks.** Flow cytometric quantification of blood **(A)** neutrophils, **(B)** B cells and **(C)** T cells at 12 weeks (n=6/group). Data were represented as mean±sem. For multiple-group comparisons, data were analysed with a Kruskal-Wallis ANOVA with Dunn's post hoc test. HFD: High fat diet.

## Supplemental Figure VII

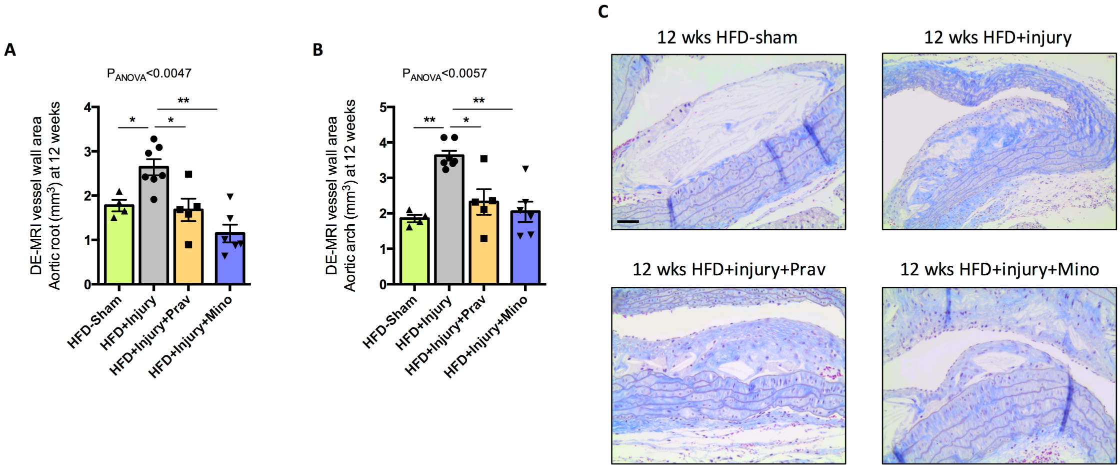


**Supplemental Figure VII. Oral administration of pravastatin and minocycline decreases plaque burden in the aortic root and aortic arch. A**, Quantification of plaque burden in the aortic root and (**B**) aortic arch as measured by the LGE-MRI images (n=4-7/group). **C**, Representative trichrome staining of the plaques located in the aortic arch in the different experimental groups. Data were represented as mean±sem. For multiple-group comparisons, data were analysed with a Kruskal-Wallis ANOVA with Dunn's post hoc test. DE: Delayed enhancement; HFD: High fat diet; Prav: Pravastatin; Mino: Minocycline; wks: weeks. *P<0.05, **P<0.01.

**Supplemental Table I. MRI parameters used to image the abdominal aorta.**

| Aorta | 3D CE-MRA | 3D DE-MRI | 3D T1 mapping |
|---|---|---|---|
| FOV (mm) | 35x35x12 | 35x35x12 | 36x22x10 |
| Resolution (mm) | 0.15x0.15x0.5 | 0.1x0.1x0.5 | 0.2x0.2x0.5 |
| TR (ms) | 28 | 27 | 9 |
| TE (ms) | 6 | 8 | 4.6 |
| Flip angle | $40^o$ | $30^o$ | $10^o$ |
| Scan time (min) | 3 | 15 | 20 |

**Supplemental Table II. MRI parameters used to image the brachiocephalic artery (BCA)**

| BCA | 3D CE-MRA | 3D DE-MRI | 3D T1 mapping |
|---|---|---|---|
| FOV (mm) | 35x35x16 | 35x35x12 | 36x22x10 |
| Resolution (mm) | 0.15x0.15x0.5 | 0.1x0.1x0.5 | 0.2x0.2x0.5 |
| TR (ms) | 28 | 27 | 9 |
| TE (ms) | 6 | 8.2 | 4.6 |
| Flip angle | $40^o$ | $30^o$ | $10^o$ |
| Scan time (min) | 3 | 15 | 20 |
